# A sex-specific reconstitution bias in the competitive CD45.1/CD45.2 congenic bone marrow transplant model

**DOI:** 10.1038/s41598-017-03784-9

**Published:** 2017-06-14

**Authors:** Salema Jafri, Stephen D. Moore, Nicholas W. Morrell, Mark L. Ormiston

**Affiliations:** 1 University of Cambridge, Department of Medicine, Cambridge, CB2 0QQ United Kingdom; 2 Queen’s University, Department of Biomedical and Molecular Sciences, Kingston, K7L 3N6 Canada

## Abstract

Allelic variants of the pan-haematopoietic cell marker CD45, identified as CD45.1 and CD45.2, have been established as a marker system to track haematopoietic cells following congenic mouse bone marrow transplants. Despite the frequent use of this model for studying the impact of genetic modifications on relative differentiation potential, it is now evident that a bias exists in CD45.1 versus CD45.2 cell reconstitution. While this bias has been demonstrated by reduced reconstitution potential in B cells of CD45.1 origin, differences in the development of other lymphocytes, as well as the impact of sex on this bias, remain uncertain. We performed bone marrow transplants with wild-type CD45.1 and CD45.2 donor cells, and characterised haematopoietic cell reconstitution in dual-expressing CD45.1/2 host mice. We report an increase in CD45.2 reconstitution in the bone marrow that persists in the spleen, thymus and blood. Through the use of CD45.1/2 hosts, we demonstrate the intrinsic bias towards CD45.2 reconstitution is independent of an immunogenic response to the CD45.1 epitope. Furthermore, we identify a sex-specific difference in reconstitution efficiencies, with female mice exhibiting a greater bias towards CD45.2 reconstitution than males. This work sheds new light on the limitations of the CD45.1/CD45.2 congenic system for tracking lymphocyte development.

## Introduction

CD45, also known as protein tyrosine phosphatase, receptor type C, is encoded by the *Ptprc* gene and is expressed on the surface of all haematopoietic cells with the exception of erythrocytes and platelets^1^. In addition to its use as a marker for cells of the haematopoietic lineage, the surface protein also acts as a costimulatory molecule for cell activation and signalling^2–4^. Different isoforms of CD45 have been identified in mice; the common form is CD45.2, which is expressed by C57BL/6 mice, and is encoded by the *Ptprc* ^*b*^ allele. However, an additional allelic variant, *Ptprc* ^*a*^, which translates to the CD45.1 form of the surface protein, has been identified in the SJL mouse strain. This CD45.1 allele has been successfully backcrossed on to the BL/6 background^5^ and has been widely used in immunological studies to track the contribution of specific genes to haematopoietic cell development^6^. In a competitive congenic bone marrow transplant model, irradiated host mice are reconstituted with a chimeric 1:1 bone marrow mix from CD45.1 and CD45.2 donors, enabling the independent tracking and quantification of reconstituted haematopoietic cells from each donor. When these assays include genetically modified mice, these animals are typically available on a BL/6 background, allowing for the identification of genetically modified cells by the CD45.2 marker when reconstituted alongside wild-type cells bearing the CD45.1 epitope.

Historically, the competitive congenic model involves the use of CD45.2^+^ BL/6 mice as wild-type hosts, due to the ease of their availability. Although CD45.1 and CD45.2 have been accepted as being functionally equivalent, this use of CD45.2 hosts raises the possibility of bias in the model secondary to alloreactivity towards the foreign CD45.1 epitope^7, 8^. There have been conflicting reports of the presence or absence of alloreactivity between CD45 epitopes^9^, depending on the study design. As demonstrated by Basu *et al*., the CD45 type of the irradiated host influences the relative reconstitution of CD45.1 and CD45.2 donor cells^10^. While the use of CD45.1 hosts and higher dose of total body gamma radiation reportedly results in lower alloreactivity^9, 11^, the presence of residual host CD45.2 or CD45.1 cells following irradiation may skew results by as much as 10% in either direction, as these cells are indistinguishable from donor-derived cells bearing the same CD45 epitope. The use of CD45.1 or CD45.2 host mice may be especially poorly-suited for studies involving the T cell lineage, as these cells are particularly resistant to irradiation, enhancing the skewing of data caused by residual host cells^12^.

Beyond these issues of alloreactivity, functional differences in the relative homing efficiency and haematopoietic cell reconstitution potential of CD45.1 and CD45.2 have also been reported in the competitive congenic model^11, 12^. These differences were originally linked to a congenic interval containing over 300 genes^11^. More recently, Mercier *et al*. identified a single exon region responsible for the difference in CD45.1 and CD45.2 reconstitution^12^. Considering the continued popularity of this model in studies of haematopoietic differentiation potential, a more complete picture of the differences between CD45.1 and CD45.2 reconstitution efficiency is required.

In the current study, we performed competitive congenic bone marrow transplants utilising CD45.1/2 host mice, thereby eliminating any potential bias associated with alloreactivity and allowing for the clear distinction of donor cells from cells of host origin. T cell, B cell and natural killer (NK) cell populations were characterized, with the goal of clarifying the intrinsic bias in development between cells of CD45.1 and CD45.2 origins in each of these subsets. Unlike previous work in the field, we present data on CD45.1 versus CD45.2 development of lymphocyte stages in both primary and secondary lymphoid tissues, including the bone marrow, thymus and spleen. Furthermore, our use of sex-matched donor-host trios allowed for the identification of a previously unappreciated sex bias in the relative reconstitution efficiency of bone marrow progenitors from CD45.1 and CD45.2 mice, with female mice demonstrating an exaggerated bias when compared to males.

## Results

### Competitively transplanted mice exhibit a CD45.2 reconstitution bias that is enhanced in females

**Figure 1:**
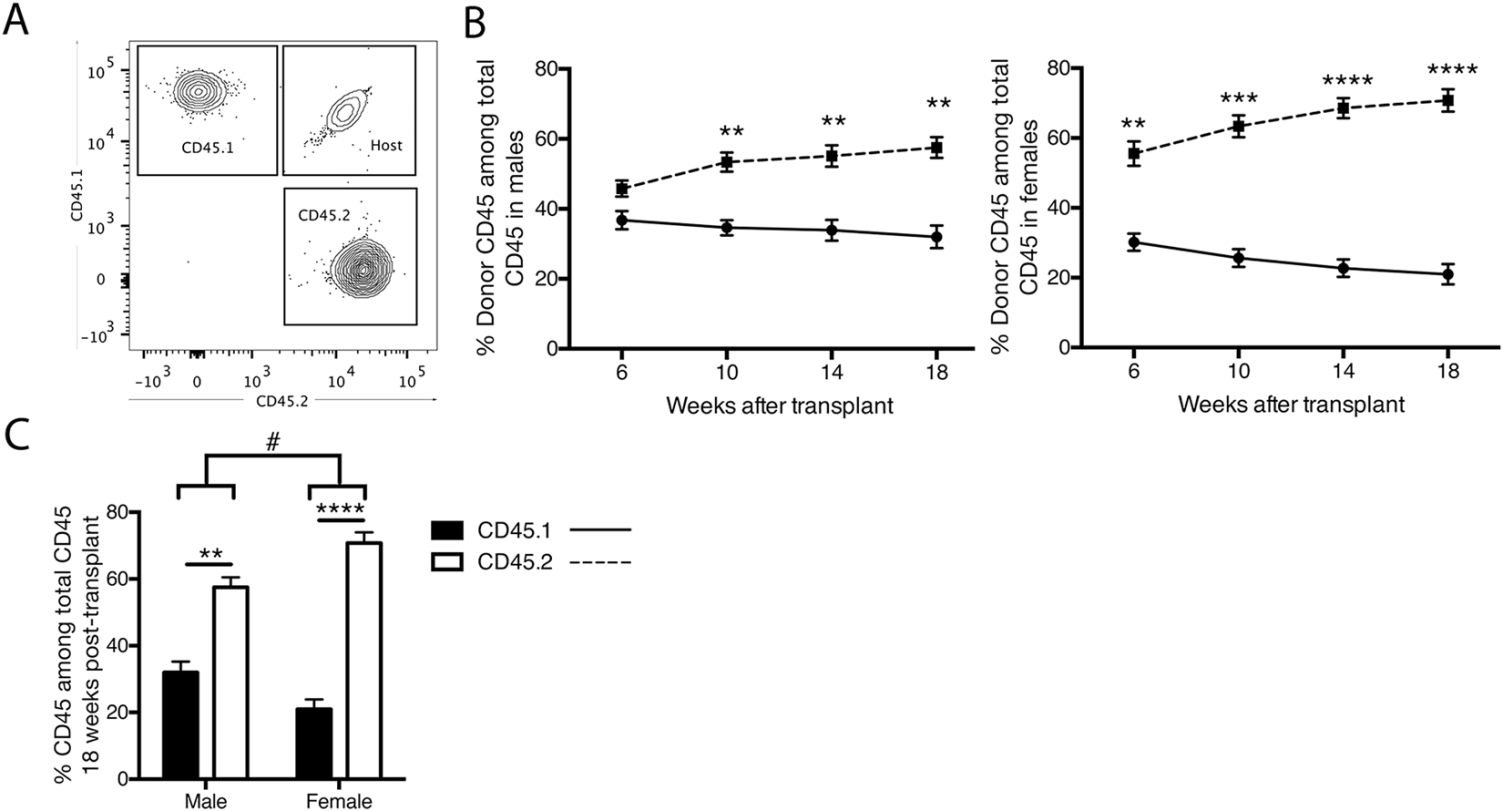
Sex bias in haematopoietic cell reconstitution by cells of CD45.1 and CD45.2 origin. (**A**) Characterisation of donor CD45.1 and CD45.2 cells in CD45.1/2 mouse blood. (**B**) Relative proportions of circulating CD45.1 and CD45.2 cells in males (left panel) and females (right panel) following bone marrow transplantation. (**C**) Proportion of circulating donor CD45 between males and females 18 weeks post-transplant. Data are presented as mean ± SEM from a total of 7–8 male mice from 4 independent transplants, and 9 female mice from 3 independent transplants. Dotted lines represent cells of CD45.2 origin, intact lines represent cells of CD45.1 origin Statistically tested with a paired t-test, **P < 0.01, ***P < 0.005, ****P < 0.001 between CD45.1 and CD45.2, ^#^P < 0.05 between males and females.

We investigated the comparative potential of cells of CD45.1 and CD45.2 origin to repopulate the lymphocytic compartments in irradiated CD45.1/2 host mice. Prior to use in transplant experiments, blood samples of donor and host mice were assessed to confirm the pan-haematopoietic expression of the CD45.1 and CD45.2 epitopes. CD45^+^ leukocytes from donor mice were found to be uniformly positive for either CD45.1 or CD45.2, whereas leukocytes from CD45.1/2 hosts were exclusively dual-expressing CD45.1^+^/CD45.2^+^ (Supplementary Figure S1). Following transplantation, a considerable increase in the reconstitution of CD45.2^+^ cells was observed in the circulation of transplanted CD45.1/2 host mice from as early as 6 weeks post-transplant in females and 10 weeks in males (Fig. 1A,B). At 18 weeks post-transplant, this bias towards the long-term reconstitution of CD45.2^+^ haematopoietic cells was greater in female mice than in males, with a 3.4-fold ratio of CD45.2^+^ to CD45.1^+^ cells in females, compared to a 1.7-fold increase in male mice (Fig. 1C). In both males and females, this enhanced reconstitution of CD45.2^+^ cells translated to increased absolute counts of CD45.2^+^ cells in all lymphocyte subsets examined, including T, B and NK cells (data not shown).

**Figure 2:**
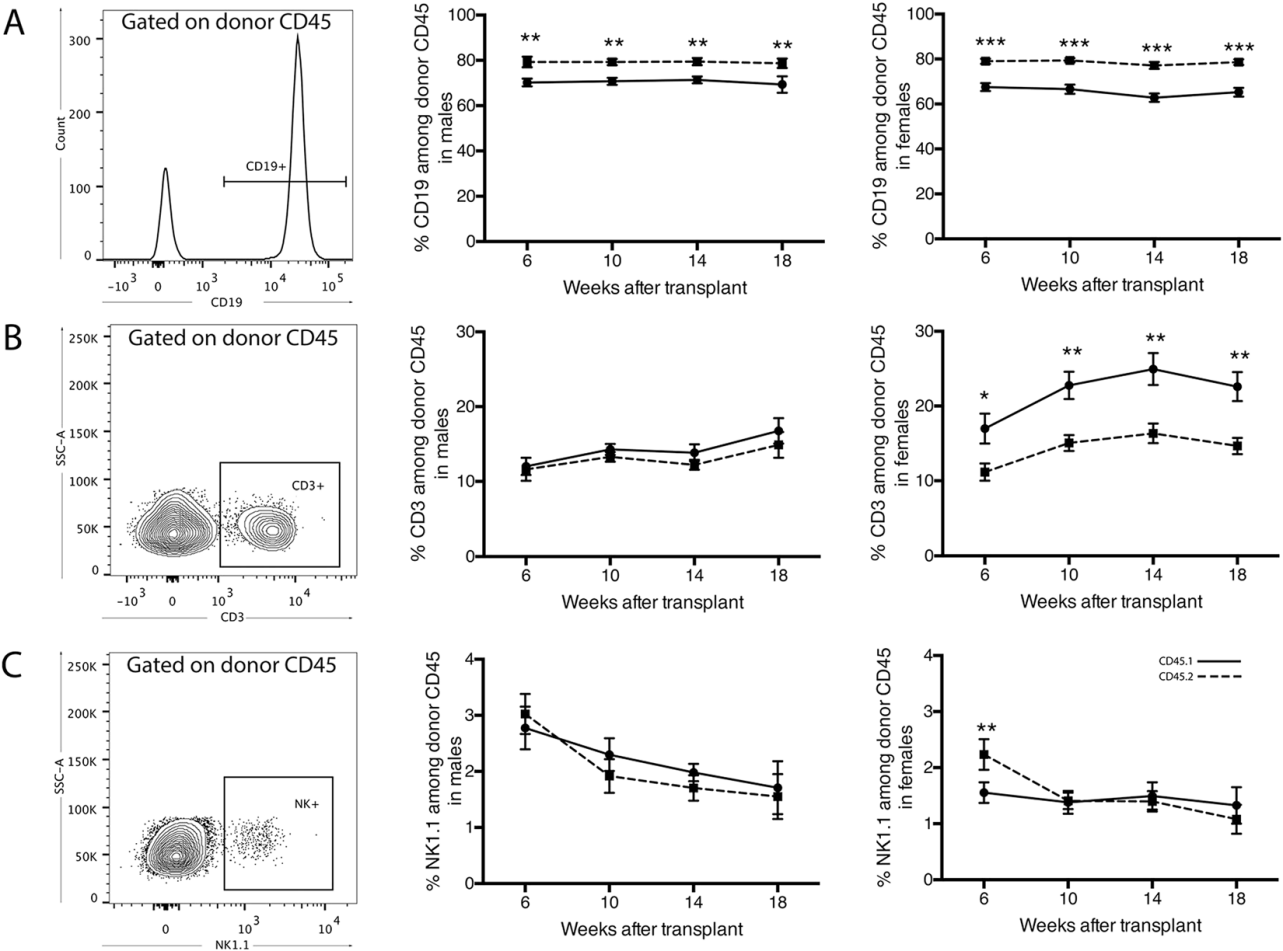
Sex bias in reconstitution of circulating lymphocytes by cells of CD45.1 and CD45.2 origin. (**A**) Characterisation of donor-specific CD19^+^ B cells, (**B**) CD3^+^ T cells and (**C**) natural killer cells in mouse blood (left panels) in males (middle panel) and females (right panel) following bone marrow transplantation. Data are presented as mean ± SEM from a total of 7–8 male mice from 4 independent transplants, and 9 female mice from 3 independent transplants. Dotted lines represent cells of CD45.2 origin, intact lines represent cells of CD45.1 origin. Statistically tested with a paired t-test, *P < 0.05, **P < 0.01, ***P < 0.005 between CD45.1 and CD45.2.

We additionally performed an examination of each lymphocyte subset as a relative proportion of donor-specific CD45^+^ cells to provide insight into the impact of donor CD45 type on the relative differentiation potential of these subsets. This analysis identified an expansion of circulating CD45.2^+^ B cells, relative to the proportion of B cells within the CD45.1^+^ population (Fig. 2A). Although a significant increase in CD45.2^+^ B cells was observed in both male and female mice (at 18-week time point P = 0.01 and 0.0002 for males and females respectively), a corresponding decrease in the relative proportion of CD45.2^+^ T cells among cells of CD45.2 origin was only seen in female mice (Fig. 2B) (P = 0.0039 at 18-week time point), alongside the more severe reconstitution bias. Despite these changes in the B cell and T cell populations, the proportion of NK cells among donor-specific CD45 cells remained unchanged in the long-term (beyond 6 weeks post-transplant), in both males and females (Fig. 2C).

### CD45.2 cells exhibit enhanced bone marrow reconstitution potential

**Figure 3:**
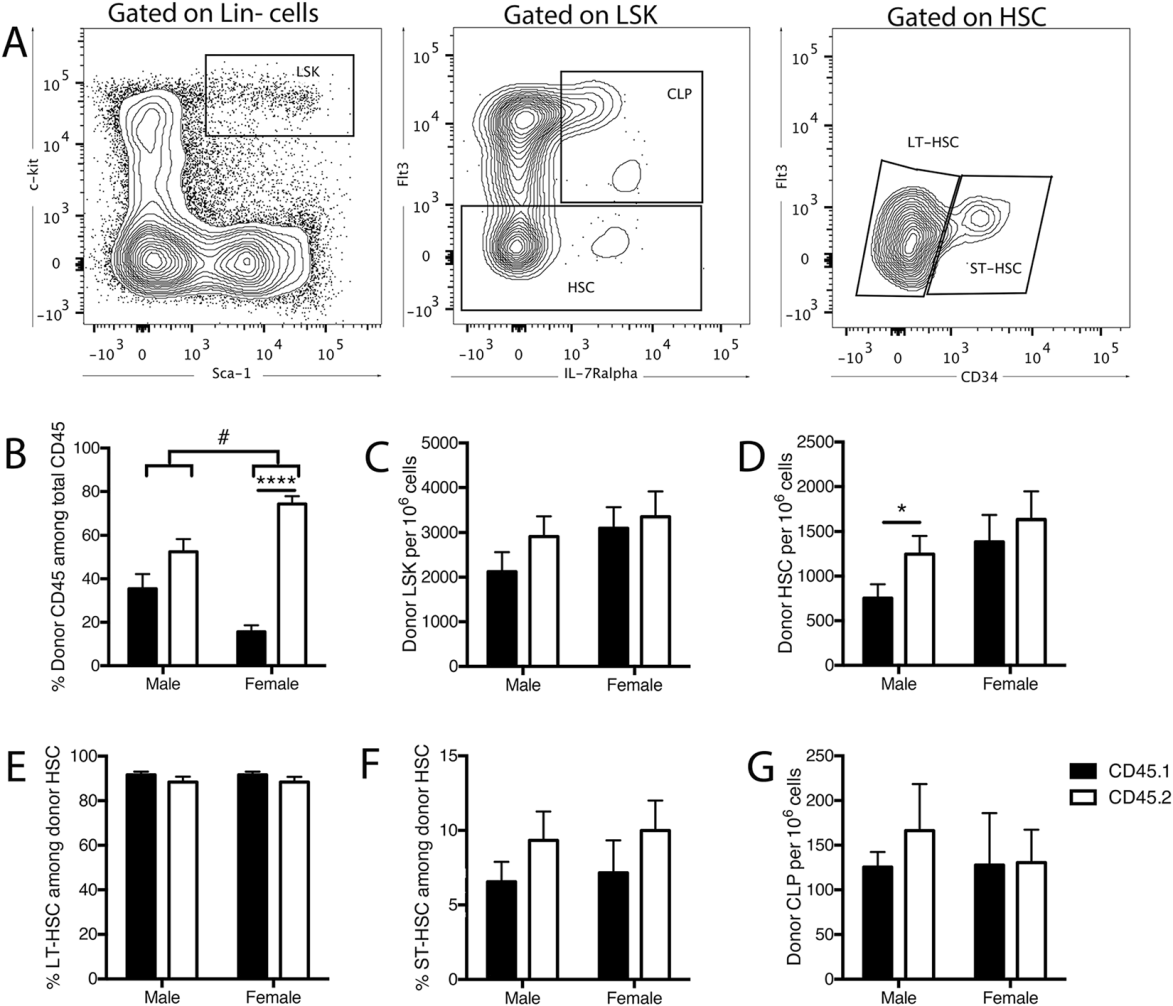
Sex bias in haematopoietic progenitor cell reconstitution by cells of CD45.1 and CD45.2 origin. (**A**) Sample flow cytometric plots depicting haematopoietic progenitor characterisation in the bone marrow of CD45.1/2 hosts 18-weeks post-bone marrow transplantation. CLP and HSC are characterised among LSK cells, long-term and short-term HSCs are characterised among Flt3^-^ HSC. (**B**) Relative proportions of CD45.1 and CD45.2 cells in the bone marrow. Proportion of donor-specific (**C**) LSK cells, (**D**) haematopoietic stem cells (HSCs), (**E**) long-term HSCs, (**F**) short-term HSCs and (**G**) common lymphoid progenitors (CLP). Data are presented as mean ± SEM from a total of 3–7 male mice from 4 independent transplants, and 4–7 female mice from 3 independent transplants. Statistically tested with a paired t-test, *P < 0.05, ****P < 0.001 between CD45.1 and CD45.2, ^#^P < 0.05 between males and females.

Following our confirmation of a CD45.2 bias in the circulation of competitively transplanted mice, an investigation of haematopoietic progenitor cell reconstitution efficiency in the bone marrow identified a dramatic increase in CD45.2^+^ cells in the bone marrow of transplanted female mice (Fig. 3A,B). Of note, a similar trend towards increased CD45.2^+^ cells was observed in male mice. However, this difference was not significant (P = 0.1696), mirroring the sex-specific differences in reconstitution bias as observed in the circulation (Figs 1 and 2). A further assessment of the relative proportions of haematopoietic progenitor cell populations revealed no differences within the bone marrow of female mice at each stage of differentiation (Fig. 3C–G). This analysis included an assessment of Lin^−^Sca1^+^c-kit^+^ (LSK) progenitor cells (Fig. 3C), which include both the CD34^−^/Flt3^−^ long-term haematopoietic stem cells (HSC; Fig. 3E) and CD34^+^/Flt3^−^ short-term HSCs (Fig. 3F), as well as the Flt3^+^IL7Rα^+^ common lymphoid progenitors (CLP; Fig. 3G). Interestingly, a significant increase (P = 0.03) in the proportion of CD45.2^+^ HSC was observed in male mice, but this did not translate further downstream to the CLP population, nor did this increase result in a significant increase (P = 0.17) of CD45.2^+^ bone marrow cells in males, relative to CD45.1^+^ cells in the same animals.

### CD45.1 versus CD45.2 bias persists in B and T lymphocyte development

**Figure 4:**
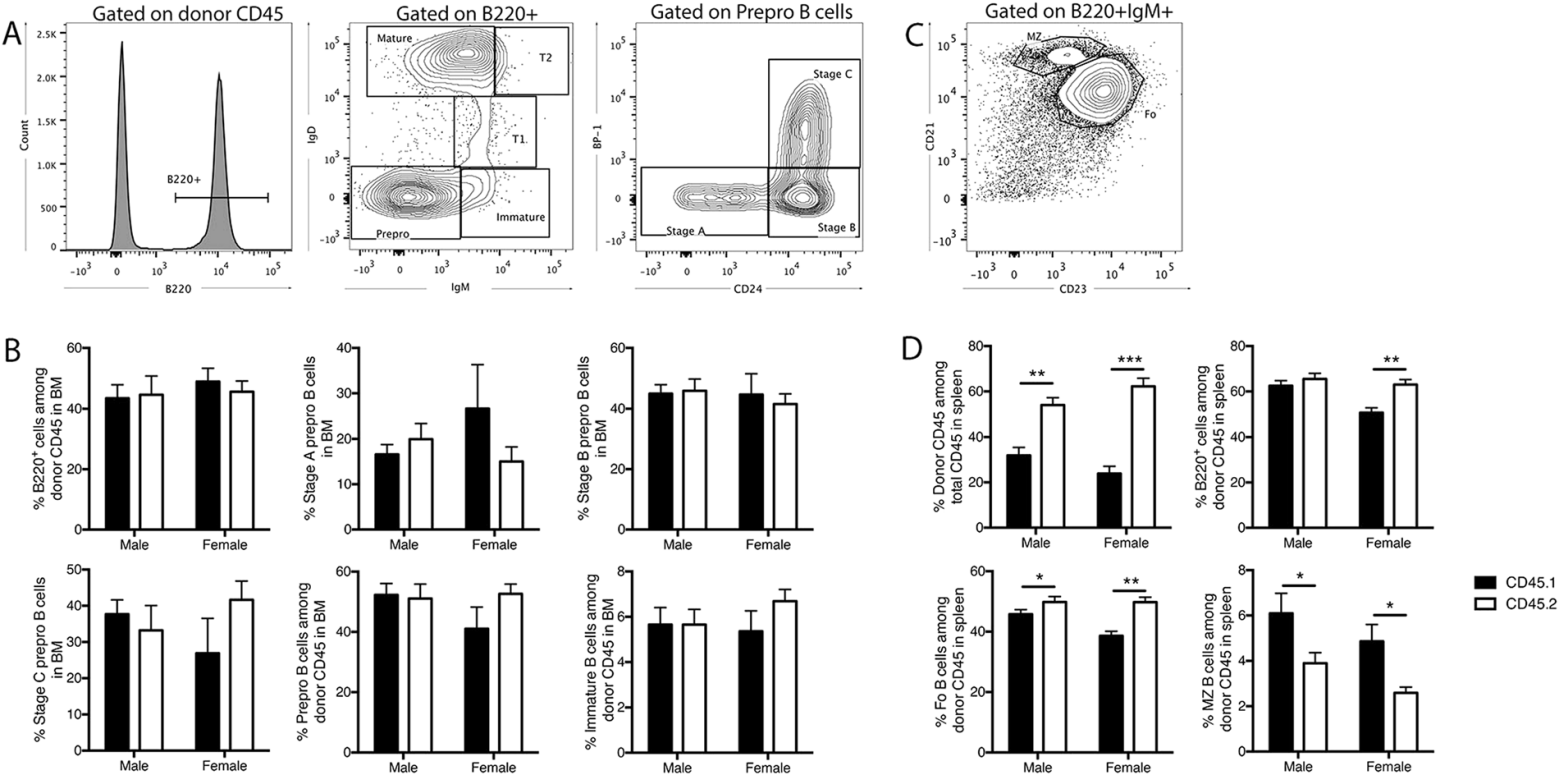
Imbalance in splenic B cell developmental stages of CD45.1 and CD45.2 origin. Sample flow cytometric plots depicting characterisation of B cell developmental stages in the (**A**) bone marrow and (**C**) spleen of CD45.1/2 hosts 18-weeks post-bone marrow transplantation. Early stage B cells at pre-pro Stages A, B and C in the bone marrow are characterised using surface expression of BP-1 and CD24 among IgD^−^IgM^−^ pre-pro B cells. (**B**,**D**) Proportion of cells originating from CD45.1 and CD45.2 donor cells at B cell developmental stages in the (**B**) bone marrow and (**D**) spleen. Data are presented as mean ± SEM from a total of 7 male mice from 4 independent transplants, and 7 female mice from 3 independent transplants. Statistically tested with a paired t-test, *P < 0.05, **P < 0.01, ***P < 0.005. BM = bone marrow, MZ = marginal zone.

Following our observation of an increased proportion of B cells among circulating lymphocytes of CD45.2 origin, we next characterized developmental stages along the B cell lineage in transplanted mice to determine the precise stage at which this B cell-specific imbalance might occur. Despite the increased absolute number of CD45.2^+^ cells in the bone marrow of transplanted mice, a comparison of early B cell developmental stages as a proportion of total CD45.1 or CD45.2 cells failed to identify a relative difference in B220^+^ B lineage cells in the bone marrow, or any of the sub-categorized B cell developmental stages identified by IgD, IgM, BP-1 and CD24 expression as per Hardy’s characterization^13^ (Fig. 4A,B). However, an imbalance in the relative proportion of CD45.2^+^ B cell subsets was observed among the more mature B cell populations characterized in the spleen (Fig. 4C,D). Follicular B cells, which form the majority population of B cells in the spleen and represent circulating peripheral B cells, were found to be increased as a proportion of total CD45.2^+^ cells. This increase in follicular B cells was accompanied by a relative reduction in the proportion of marginal zone (MZ) B cells among cells of CD45.2 donor origin, which tend to remain resident in the marginal zone of the spleen.

**Figure 5:**
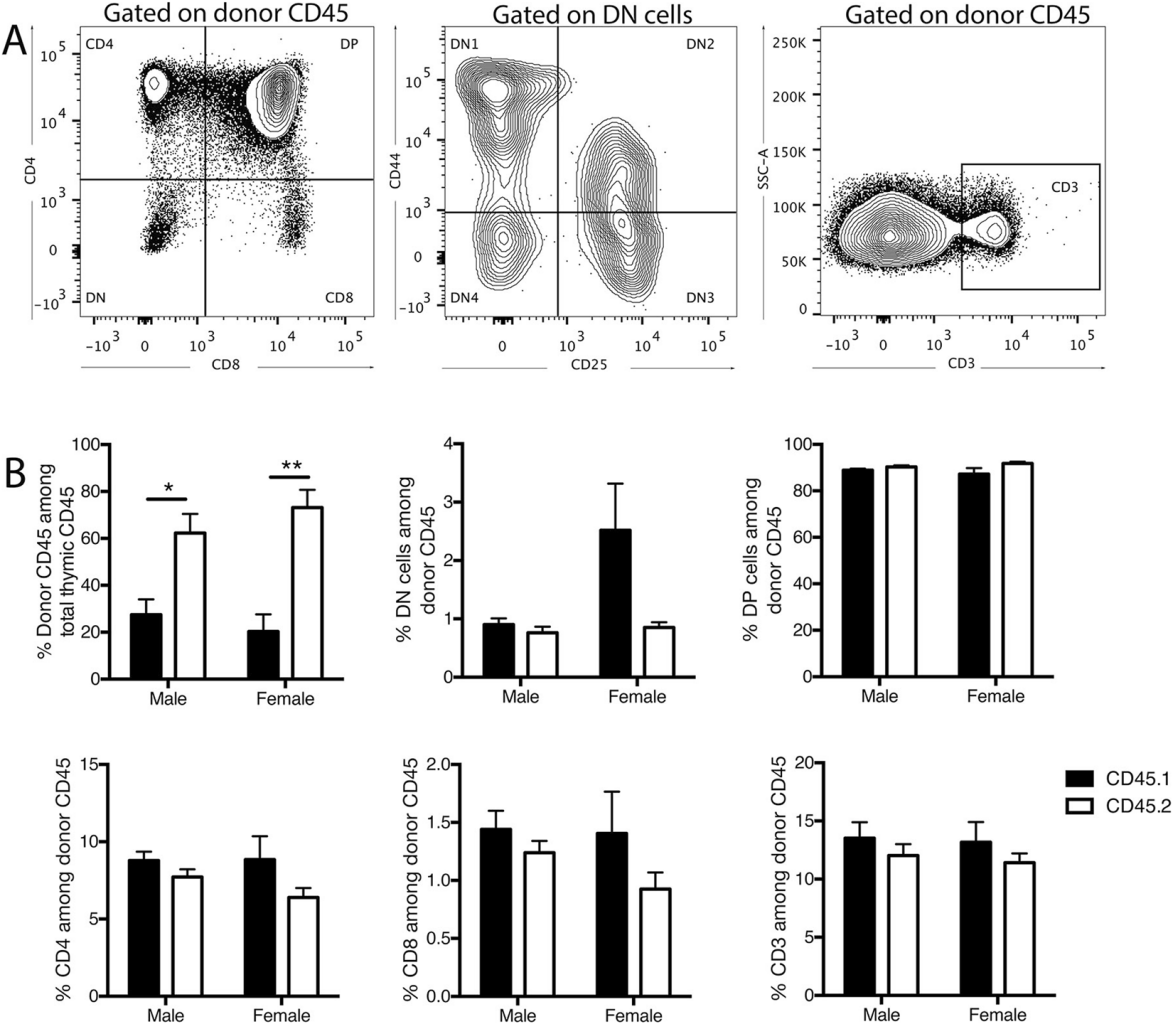
Increased cells of CD45.2 origin compared to CD45.1 origin in thymus with no influence on individual T cell developmental stages. (**A**) Sample flow cytometric plots depicting characterisation of T cell developmental stages in the thymus of CD45.1/2 hosts 18-weeks post-bone marrow transplantation. Early double negative stages (DN1–DN4) are characterised based on surface expression CD4 and CD8 among CD4^−^CD8^−^ (DN) cells. (**B**) Proportion of cells originating from CD45.1 and CD45.2 donor cells at T cell developmental stages in the thymus. Data are presented as mean ± SEM from a total of 7 male mice from 4 independent transplants, and 7 female mice from 3 independent transplants. Statistically tested with a paired t-test, *P < 0.05, **P < 0.01. DN = double negative, CD4^−^CD8^−^. DP = double positive CD4^+^CD8^+^.

The potential influence of the CD45 variants on T cell development was also assessed through a cytometric analysis of the various stages of thymocyte development (Fig. 5A). As with the bone marrow, spleen and blood, analysis of thymocytes revealed an increased proportion of cells of CD45.2 origin compared to CD45.1 in both male and female mice that appeared to be more pronounced in females (Fig. 5B). However, as with our assessment of B cell development in the bone marrow, an examination of the proportions of T cells at each developmental stage among cells of CD45.1 and CD45.2 origin revealed no significant differences, suggesting no specific influence of these allelic variants on the efficiency of T cell development in the thymus.

## Discussion

We report that cells of CD45.1 and CD45.2 origins are intrinsically non-identical in their lymphocyte development potential following bone marrow transplant into CD45.1/2 hosts. We further show that the degree of this bias is different between male and female mice, with female mice showing a more dramatic difference in CD45.2 versus CD45.1 reconstitution potential than males. This sex-specific difference is primarily evident among circulating lymphocyte populations, which exhibit the greatest CD45.2 versus CD45.1 reconstitution bias. Additionally, among the increased CD45.2 cells, we have observed a preferential expansion of CD45.2^+^ B cells in females that results in a relative reduction in the proportion of circulating T cells. This skewing of T lymphocyte proportion is not observed to a significant degree in male mice.

Our data builds on previous findings by Waterstrat and colleagues, as well as Basu and colleagues, both of which reported a differential capacity of CD45.1 and CD45.2 cells to reconstitute transplanted mice^10, 11^. Earlier studies examining this issue used host mice of CD45.2 origin, which have the potential to skew results through the persistence of residual host cells following irradiation. The use of CD45.2 hosts may also influence results through immunogenic responses, resulting in increased CD45.2 cell repopulation. In the current study, this issue of immunogenicity was addressed through the use of dual-expressing CD45.1/2 mice as hosts.

Interestingly, the bias between CD45.1 and CD45.2 cells was observed as early as 6 to 10 weeks following bone marrow transplants, and persisted through the 18-week long-term post-transplant period. Although the bias in our studies manifested earlier than has been reported in some previous reports^10, 11^, our results are in line with work by Mercier and colleagues, who demonstrated an early reconstitution bias in studies that made use of exclusively female mice^12^. These discrepancies between published works may be linked to subtle differences in the protocols used, including the irradiation dose and the number of transplanted cells. Both groups showing a CD45.1 versus CD45.2 bias exclusively at later time points used a single dose of irradiation and transplanted a higher number of bone marrow cells (4 × 10^6^ and 5 × 10^6^ cells) into host mice^10, 11^. In comparison, our study and that described by Mercier and colleagues, used two doses of irradiation followed by injection of 1 × 10^6^ cells into irradiated hosts. Of note, the study by Mercier and colleagues also includes CD45.1 mice bearing a correction of the bias-causing region of the genome^12^. While these animals represent an improved control for competitive bone marrow transplants, our findings indicate that a thorough examination of any potential sex bias in this new model may be required.

Our assessment of the stages of haematopoietic progenitor cell development in the bone marrow failed to identify significant differences in the relative proportion of any particular haematopoietic progenitor cell population. This finding suggests that a large part of the observed CD45.2 versus CD45.1 reconstitution bias originates at the earliest stage of haematopoiesis and is likely a result of differential homing of donor cells with long-term repopulation potential to the bone marrow. Similarly, the populations representing the stages of B cell development in the bone marrow and T cell development in the thymus were found to be proportionally equivalent between cells of CD45.1 and CD45.2 origins, despite the overall increase in CD45.2 reconstitution. In contrast, the overall bias towards CD45.2^+^ cells in the circulation was enhanced by what appears to be a direct effect of the CD45 variant on the differentiation of mature B cell populations in the spleen. This difference was demonstrated by the increased proportion of follicular B cells and relative reduction in marginal zone B cells among cells of CD45.2 origin. These findings in the spleen help to explain our observation of proportionally increased B cells in the circulation, as splenic follicular B cells represent the population of B cells that enter the blood stream.

In summary, our report of this sex bias in the competitive congenic transplant model provides new insight into this experimental system and helps to clarify the specific stages of reconstitution that are impacted by variation in CD45 alleles. Of note, the current study was primarily focused on the impact of this transplant model on lymphocyte subsets, including T, B and NK cells. Although an assessment of myeloid cell types was not included in the current study, a similar strategy to the one shown here could be used to examine the impact of this model on specific myeloid subsets. This work also highlights the need for appropriate controls and careful analysis in the execution of congenic bone marrow transplant experiments. Specifically, control transplants with wild-type donor cells of both CD45.1 and CD45.2 origins are required, against which transplants using CD45.2 donor cells from genetically modified mice can be normalized. As the bias toward CD45.2 would be present in both test and control animals, calculation of the impact of specific genetic manipulations on haematopoietic potential can be determined relative to these wild-type only controls. Unfortunately, such controls are not a consistent feature of all studies making use of this transplant model. Without these controls, this system of bone marrow cell transplantation is unsuitable to study the effect of specific genetic manipulations on T and B cell development, due to the intrinsic reconstitution bias between CD45.1 and CD45.2 donors. It is notable that the NK cell subset is least influenced by the use of the congenic CD45 marker system, particularly in studies utilizing male mice, as the long-term repopulation efficiency of these cells as a proportion of donor-matched CD45^+^ cells remains unaltered in both male and female models.

## Methods

### Mice

Wild-type CD45.1 mice on a BL/6 background were kindly provided by Prof Tony Green at the University of Cambridge. Wild-type CD45.2 mice were C57BL/6 mice directly purchased from Charles River Laboratories (Wilmington, MA). CD45.1 mice were bred with C57BL/6 mice to generate CD45.1/2 mice expressing both the allelic variants. Mice were housed in individually ventilated cages at Central Biomedical Services, and provided food and water ad libitum.

All studies were approved by the local animal facility, Central Biomedical Services, University of Cambridge, and carried out in accordance with the UK Animals (Scientific Procedures) Act 1986 under Project Licence 80/2460 approved by the UK Home Office. All procedures were performed by personnel holding a procedure personal licence. Young adult mice between 6 to 10 weeks of age were sex- and age-matched for bone marrow transplants.

### Bone marrow transplants

CD45.1/2 host mice were administered total body gamma radiation with two doses of 5 Gy from a caesium source, three hours apart. Donor CD45.1 and CD45.2 wild-type mice were sacrificed by dislocation of the neck and femurs were harvested and collected in ice-cold phosphate buffered saline (PBS). Bone marrow cells were isolated in a sterile environment by flushing the femur with a 23 G needle using PBS + 2% Fetal Bovine Serum. Harvested cells were filtered through a 40 μm cell filter and enumerated. Donor mixes were prepared by mixing CD45.1 and CD45.2 bone marrow cells in a 1:1 ratio and resuspended in sterile PBS. The donor mix was validated to have an equal proportion of CD45.1 and CD45.2 cells by flow cytometric analysis. A total of 1 × 10^6^ nucleated bone marrow cells in a final volume of 200 μL was transplanted into each irradiated host mouse via the tail vein. Host mice were administered oral antibiotic, Baytril (Bayer, Leverkusen, Germany), as a prophylactic against bacterial infections, in drinking water for two weeks post-transplant. Transplanted hosts were bled via the saphenous vein 6-, 10-, 14- and 18 weeks post-transplant to characterise circulating lymphocyte populations. Mice were sacrificed 18 weeks post-transplant and bone marrow, thymus and spleen were harvested for characterisation of lymphocyte development stages.

### Flow cytometry

Single cell suspensions were created by passing bone marrow cells, crushed spleen, and crushed thymus through 40 μm cell filters. Following red blood cell lysis, up to 1 × 10^6^ cells per sample were blocked with FcγIII/ II receptor antibody (clone 2.492; BD Pharmingen, Franklin Lakes, NJ). Blocked samples were stained with antibody mixes prepared in staining buffer (0.5% bovine serum albumin and 2 mM EDTA in PBS) in a 30-minute incubation in the dark at 4 °C. All antibodies were sourced from Biolegend, San Diego, CA, unless stated otherwise.

Anti-CD45.1-FITC (clone A20) and anti-CD45.2-Alexa Fluor 700 (clone 104) were used to characterise cells of donor and host origins. Blood was stained with anti-CD19-PE (clone 6D5), anti-CD3-APCEF780 (clone 17A2; eBioscience, Affymetrix, Santa Clara, CA), and anti-NK1.1-Pacific Blue (clone PK136) to characterize circulating B cells, T cells and NK cells respectively. Haematopoietic progenitor cell populations in the bone marrow were characterized with anti-c-kit-APC (clone 2B8), anti-Sca1-APCCy7 (clone D7), anti-Flt-3-PE (clone A2F10), anti-IL-7Rα-PECy7 (clone A7R34), anti-CD34-PerCPCy5.5 (clone HM34) and a lineage cocktail antibody conjugated to Pacific Blue specific for Ter119 (clone Ter-119), Gr-1 (clone RB6-8C5), CD3 (clone 17A2), CD19 (clone 6D5), NK1.1 (clone PK136) and CD11b (clone M1/70).

Bone marrow cells were also stained for early B cell developmental markers based on Hardy’s characteristion^13^: anti-B220-Pacific Blue (clone RA3-6B2), anti-CD24-PECy7 (clone MI/69), anti-BP-1-PE (clone 6C3), anti-IgD-APC (clone 11–26 c.2a) and anti-IgM-APCCy7 (clone RMM-1). A separate panel was designed to characterize splenic B cell subsets of marginal zone and follicular cells with anti-B220-Pacific Blue (clone RA3-6B2), anti-IgM-APCCy7 (clone RMM-1), anti-CD21-PE (clone 7E9) and anti-CD23-PECy7 (clone B3B4).

Thymic cells were stained for T-cell developmental markers: anti-CD3-APCEF780 (clone 17A2; eBioscience, Affymetrix, Santa Clara, CA), anti-CD4-PE (clone GK1.5), anti-CD8-Pacific Blue (clone 53–6.7), anti-CD44-APC (clone IM7) and anti-CD25-PerCPCy5.5 (clone 3C7).

Live stained cells were analysed on a Fortessa LSR flow cytometer (BD Biosciences, Franklin Lakes, NJ) at the NIHR Cambridge BRC Cell Phenotyping Hub and data were later anlaysed using FlowJo version 10.0.6.

### Statistical analysis

All data were collated in GraphPad Prism 6 which was also used for statistical analysis. All data are presented as mean ± standard error of the mean (SEM). A paired two-tailed t-test was performed to determine the level of significant difference between donor cells of CD45.1 and CD45.2 origins. A two-way ANOVA with Bonferroni post-hoc test was used to determine differences between males and females. A probability (P)-value < 0.05 was considered significant.

A total of 9 mice were assigned to each group, and 3–4 transplants were performed to achieve this number based on litter sizes. However, during the course of the experiment some males were lost due to fighting wounds and did not make it to the 18-week end-point therefore altering sample sizes. Sex-matched littermates were randomly assigned to each transplant group. Bone marrow transplants and data collection by flow cytometry were performed in a double-blinded fashion.

## Electronic supplementary material

Supplementary Figure S1
