# Supplementary material for: A sex-specific reconstitution bias in the competitive CD45.1/CD45.2 congenic bone marrow transplant model
Source: Sci Rep. 2017 Jun 14;7:3495. doi: 10.1038/s41598-017-03784-9 (PMC5471196; doi:10.1038/s41598-017-03784-9)
Supplement: Supplementary file 1 — Supplementary Figure S1 [file 41598_2017_3784_MOESM1_ESM.pdf]

Online supplementary information

A sex-specific reconstitution bias in the competitive CD45.1/CD45.2 congenic bone marrow transplant model

Salema Jafri, Stephen D Moore, Nicholas W Morrell & Mark L Ormiston

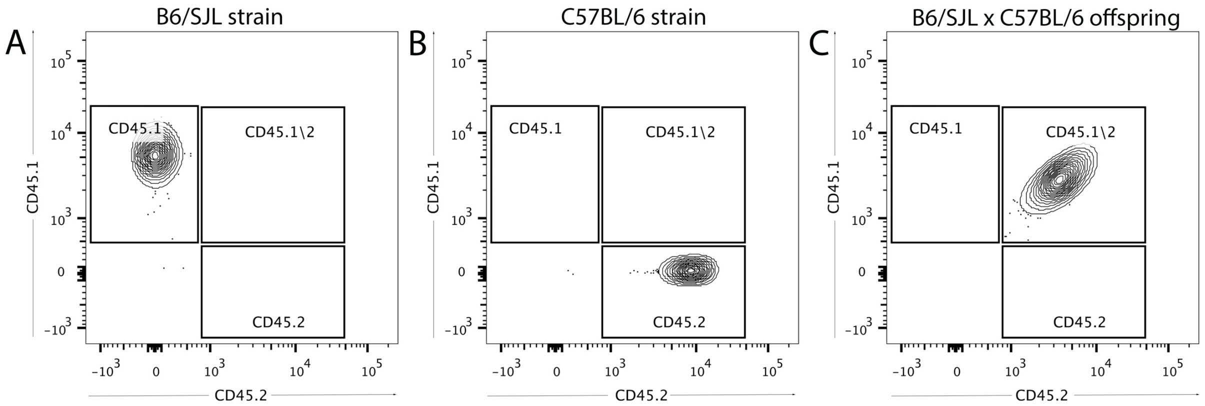

Supplementary figure S1: Peripheral blood staining of CD45 epitopes in mouse strains. Flow cytometric plots showing (A) blood cells from the B6/SJL mouse strain singly express CD45.1, (B) blood cells from the C57BL/6 strain singly express CD45.2, and (C) offspring of the B6/SJL and C57BL/6 mouse strains have blood cells doubly expressing CD45.1/2 without any singly expressed epitopes.
